# PReS-FINAL-2328: Developing and validating the new severity score for FMF

**DOI:** 10.1186/1546-0096-11-S2-P318

**Published:** 2013-12-05

**Authors:** E Demirkaya, C Acikel, A Gul, M Gattorno, E Ben-Chetrit, H Ozdogan, P Hashkes, A Polat, A Livneh, S Ozen

**Affiliations:** 1 FMF Arthritis Vasculitis and Orphan disease Research in Pediatric Rheumatology (FAVOR), ankara, Turkey

## Introduction

One of the main features, in the follow-up of a patient with a chronic disorder is severity. Definition in severity is quite difficult since the disease manifests with various clinical and laboratory features. On the other hand, there have been many articles reported in literature about the lack of consistency between currently used severity scores such as Mor, Pras and Tel Hashomer criteria.

## Objectives

The aim of this study was to develop and validate a new set of criteria for the assessment of disease severity for FMF patients by a multicenter study.

## Methods

After three rounds of Delphi survey, an elite expert panel that consisted of ten rheumatologists and one facilitator gathered in a consensus meeting in November 2012. The candidate criteria which were defined after Delphi rounds were discussed in the consensus meeting, potential criteria set were purified and the description of each item was ascertained. In order to evaluate the validity of criteria set, each expert brought together the data of clinical, laboratory manifestations and physician's global assessments of 10-50 patients from their center. Logistic regression analysis was used to evaluate predictable value of each item and ROC curve analysis was performed to demonstrate the success of the criteria set.

## Results

In expert panel nine items were selected as the identifiers of severity. Area under the curve (AUC) was calculated as 0,825 for this criteria set. When each item was evaluated individually, renal failure, nephrotic proteinuria and mean duration of episodes had the least contribution to prediction of severity. Besides, in logistic regression analysis these criteria were eliminated from the terminal modal. After reducing the criteria set, AUC was calculated as 0,808. Additional analysis demonstrated that the reduced model was as successful as full model. AUC was higher than 0,75 in both pediatric and adult patient groups and the results were better in adult group.

## Conclusion

Initial validation analysis showed that the scale could be used in assessing severity in FMF patients. The validation of the criteria should be performed on FMF patients attending outpatient clinics in routine visits.

## Disclosure of interest

None declared.

